# Functional genomics of fuzzless-lintless mutant of *Gossypium hirsutum* L. cv. MCU5 reveal key genes and pathways involved in cotton fibre initiation and elongation

**DOI:** 10.1186/1471-2164-13-624

**Published:** 2012-11-14

**Authors:** Kethireddy Venkata Padmalatha, Deepak P Patil, Krishan Kumar, Gurusamy Dhandapani, Mogilicherla Kanakachari, Mullapudi LV Phanindra, Saravanan Kumar, T C Mohan, Neha Jain, Arkalgud H Prakash, Hiremath Vamadevaiah, Ishwarappa S Katageri, Sadhu Leelavathi, Malireddy K Reddy, Polumetla Ananda Kumar, Vanga Siva Reddy

**Affiliations:** 1 National Research Centre on Plant Biotechnology, Pusa Campus, New Delhi, 110 012, India; 2 Plant Transformation Group, International Center for Genetic Engineering and Biotechnology, Aruna Asaf Ali Marg, New Delhi, 110067, India; 3 Agricultural Research Station, Dharwad Farm, University of Agricultural Sciences, Dharwad, India; 4 Central Institute for Cotton Research, Regional Station, Coimbatore, Tamil Nadu, India

## Abstract

**Background:**

Fuzzless-lintless cotton mutants are considered to be the ideal material to understand the molecular mechanisms involved in fibre cell development. Although there are few reports on transcriptome and proteome analyses in cotton at fibre initiation and elongation stages, there is no comprehensive comparative transcriptome analysis of fibre-bearing and fuzzless-lintless cotton ovules covering fibre initiation to secondary cell wall (SCW) synthesis stages. In the present study, a comparative transcriptome analysis was carried out using *G*. *hirsutum* L. cv. MCU5 wild-type (WT) and it’s near isogenic fuzzless-lintless (*fl*) mutant at fibre initiation (0 dpa/days post anthesis), elongation (5, 10 and 15 dpa) and SCW synthesis (20 dpa) stages.

**Results:**

Scanning electron microscopy study revealed the delay in the initiation of fibre cells and lack of any further development after 2 dpa in the *fl* mutant. Transcriptome analysis showed major down regulation of transcripts (90%) at fibre initiation and early elongation (5 dpa) stages in the *fl* mutant. Majority of the down regulated transcripts at fibre initiation stage in the *fl* mutant represent calcium and phytohormone mediated signal transduction pathways, biosynthesis of auxin and ethylene and stress responsive transcription factors (TFs). Further, transcripts involved in carbohydrate and lipid metabolisms, mitochondrial electron transport system (mETS) and cell wall loosening and elongation were highly down-regulated at fibre elongation stage (5–15 dpa) in the *fl* mutant. In addition, cellulose synthases and sucrose synthase C were down-regulated at SCW biosynthesis stage (15–20 dpa). Interestingly, some of the transcripts (~50%) involved in phytohormone signalling and stress responsive transcription factors that were up-regulated at fibre initiation stage in the WT were found to be up-regulated at much later stage (15 dpa) in *fl* mutant.

**Conclusions:**

Comparative transcriptome analysis of WT and its near isogenic *fl* mutant revealed key genes and pathways involved at various stages of fibre development. Our data implicated the significant role of mitochondria mediated energy metabolism during fibre elongation process. The delayed expression of genes involved in phytohormone signalling and stress responsive TFs in the *fl* mutant suggests the need for a coordinated expression of regulatory mechanisms in fibre cell initiation and differentiation.

## Background

Cotton is a commercially important fibre crop and is used as a major source of natural textile fibre and cottonseed oil. Among the four cultivated species, *Gossypium hirsutum* represents over 95% of the cultivated cotton worldwide whereas the other three species, *G*. *barbadense*, *G*. *arboreum* and *G*. *herbaceum* together represent the remaining 5%. Cotton fibres are single-celled seed trichomes that develop from the ovule epidermal cells. About 30% of the seed epidermal cells differentiate into spinnable fibres [1,2]. Cotton fibre development includes four distinct, but overlapping stages: initiation, elongation/primary cell wall (PCW) synthesis, secondary cell wall (SCW) synthesis and maturation. The fibre cell initiation usually occurs from 2–3 days before anthesis to 2–3 days post anthesis (dpa) and fibre cell elongation occurs up to 20 dpa. However, fast elongation of fibre cell occurs between 5 to 15 dpa. Secondary cell wall synthesis starts at about 20 dpa and continues up to 45 dpa. During this period large amount of cellulose (>90%) deposition takes place and the fibre cell wall becomes thick. In the final maturation stage (45–50 dpa) fibres undergo dehydration and produce mature cotton lint [1-3].

Cotton fibre is considered as an excellent single-celled model system for studying the molecular mechanisms controlling the plant cell initiation, elongation and secondary cell wall biosynthesis. In recent years, functional genomics-based approaches have been widely used to investigate the genes involved in cotton fibre development [2-8]. Phytohormones such as ethylene [9], auxins [10,11] and brassinosteroids (BR) [12,13] and transcription factors such as MYB25 [14] and MYB25-like [15,16] were shown to be involved in fibre development. Cotton fibre elongates by diffusion growth mechanism and the turgor driven force is required for unidirectional elongation [17,18]. Therefore, osmotically active solutes (soluble sugars, potassium and malate) and ion-transporters (H^+^-ATPases and K^+^-transporter) play an important role in maintaining the osmotic potential of the elongating fibre cell [18]. It is reported that the closure of plasmodesmata (PD) and the coordinated up-regulation of potassium (K^+^) and sugar transporters during fibre elongation stage maintains the turgor pressure required for the fibre cell elongation and the duration of PD closure correlates positively with the fibre length [19]. In addition, it has been reported that ROS (reactive oxygen species) homeostasis is the central regulatory mechanism for cotton fibre initiation and differentiation [8]. Carbohydrate and energy metabolisms play an important role in the fibre development by providing the carbon skeletons for the synthesis of cell wall polysaccharides and fatty acids [5,7,20,21]. Several studies have shown the role of xyloglucan and pectin modifying enzymes [22], arabinogalactans [23] and expansins [24] in cell wall loosening and expansion during fibre elongation stage. Further actin cytoskeleton plays an important role during fibre elongation stage and reorientation of cytoskeleton microtubules is required for the onset of secondary cell wall synthesis [25-27]. Despite extensive research on cotton fibre biology over the last few decades, the mechanisms controlling fibre development remain largely unknown.

The fuzzless-lintless (*fl*) ovules of cotton mutant are ideal material for identifying genes involved in the fibre development through comparative approaches. A few comparative transcriptome and proteome studies were carried out to understand the genes involved in fibre initiation and differentiation using the ovules of fuzzless-lintless mutant and its wild-type [4,8,28]. Similarly, a comparative proteome study was carried out using ovules of *fl* mutant and its wild-type to identify the differentially expressed proteins at elongation stage [7]. However, comprehensive transcriptome studies employing fuzzless-lintless mutants involving all the important stages (initiation, elongation and secondary cell wall synthesis) of fibre development are not yet reported.

In the present study, comparative transcriptome analysis of *fl* mutant with its wild-type (WT), *G*. *hirsutum* L. cv. MCU5 at fibre initiation (0 dpa), elongation (5, 10 and 15 dpa) and SCW synthesis stage (20 dpa) was carried out using Affymetrix cotton GeneChip genome array. Data from this study suggests that stress responsive transcription factors and the genes involved in calcium (Ca^2+^) and phytohormone-mediated signalling pathways play a crucial regulatory role in fibre cell initiation and differentiation. Our study also revealed the down-regulation of several genes involved in interconversion of sugar molecules and mitochondrial electron transport system (mETS) that are required for the synthesis of cell wall polysaccharides and fatty acids and maintenance of redox-homeostasis during fibre elongation. The comprehensive transcriptome analysis identified several stage specific genes and pathways operating during the fibre development that might be useful for the improvement of cotton fibre.

## Results and discussion

### Morphology of fuzzless-lintless mutant

**Figure 1:**
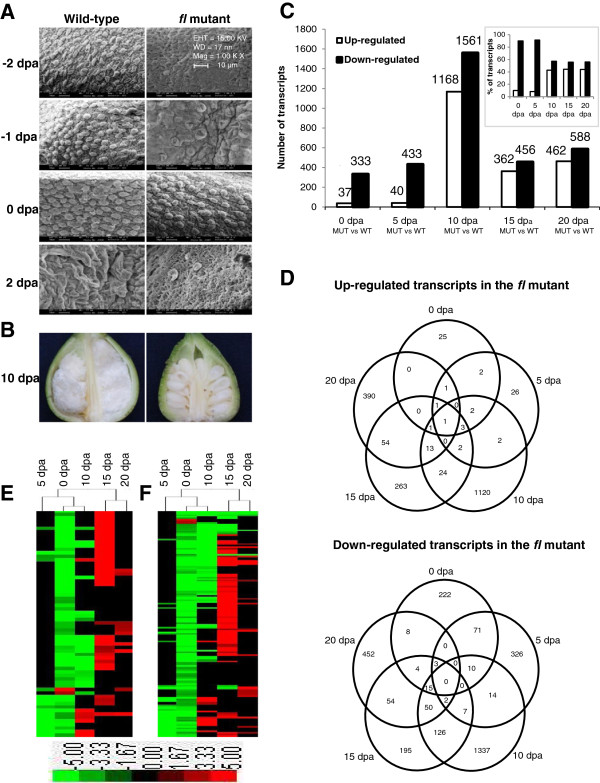
**Scanning electron microscopy (SEM) and transcriptome analyses during fibre development stages in *G*. *hirsutum *L. cv. MCU5 wild-type and its near isogenic fuzzless-lintless (*fl*) mutant.** (**A**) SEM images (bar is 10 μM) of epidermal layer of −2 to 2 dpa ovules from the WT and *fl* mutant. (**B**) Fibre-bearing ovules of WT and fuzzless-lintless ovule of mutant at 10 dpa. (**C**) Number of differentially expressed transcripts (DETs) during fibre development stages in the *fl* mutant as compared to their respective stages in WT and inset represents the percentage of up- and down-regulated transcripts in the *fl* mutant (**D**) Venn diagrams showing the commonly up- and down-regulated transcripts among the fibre development stages in the *fl* mutant. Cluster analysis showing the down-regulation of transcripts related to transcription factors (**E**) and phytohormones (**F**) at 0 dpa and up-regulation of about 50% of those transcripts at 15 dpa. DETs with *p* value ≤ 0.01 and fold change ≥ 3 were included and presented in Additional file 1.

The near isogenic *fl* mutant employed in this study is a spontaneous mutant of *G*. *hirsutum* L. cv. MCU5 first identified in 1984 [29] and maintained as a pure fuzzless-lintless line in the germplasm collections at the Cotton Breeding Station, Tamil Nadu Agricultural University, Coimbatore [29,30]. Morphological and growth parameters of the *fl* mutant including plant height, leaf size, flower colour, number of bolls per plant, number of seeds per boll, etc. are very similar to that of WT. Scanning electron microscopy (SEM) study was carried out to identify the differences in early stages of fibre development by comparing the ovule of *fl* mutant with that of WT (Figure 1A). SEM analysis revealed the presence of fibre cell initials in the *fl* mutant, though very less in numbers as compared to WT. Fibre initials could be seen two days before anthesis (−2 dpa) in the WT and were more prominent at 0 dpa. Similarly, the fibre initials were also observed on the *fl* mutant, though not very prominent at −2 dpa, but were more visible at 0 dpa (Figure 1A). This suggests the probable delay of fibre initiation in the *fl* mutant. However, further growth of fibre initials were not observed in the *fl* mutant leading to its fuzzless-lintless status (Figure 1A and B).

### Comparative transcriptome analysis

Comparative transcriptome analysis was carried out at fibre initiation (0 dpa), elongation (5, 10 and 15 dpa) and SCW synthesis (20 dpa) stages using ovules of *fl* mutant and fibre-bearing ovules of WT to decipher molecular mechanisms involved in fibre cell development. Affymetrix cotton GeneChip genome arrays were used for transcriptome analysis. Transcripts with false discovery rate (FDR) adjusted *p* value ≤ 0.01 and fold change of  ≥ 3.0 were considered as differentially expressed transcripts (DETs) in the *fl* mutant as compared to their respective stages in WT (Figure 1C, Additional file 1). The number of down-regulated transcripts was more as compared to up-regulated transcripts in various stages analysed. Further, the percentage of down-regulated transcripts was very high at fibre initiation (0 dpa, 90.0%) and early elongation (5 dpa, 91.5%) stages as compared to the later stages (Figure 1C). Data analysis revealed that majority of the DETs showed stage specific expression pattern with minor overlap among the stages (Figure 1D).

### Annotation and functional classification of DETs

**Figure 2:**
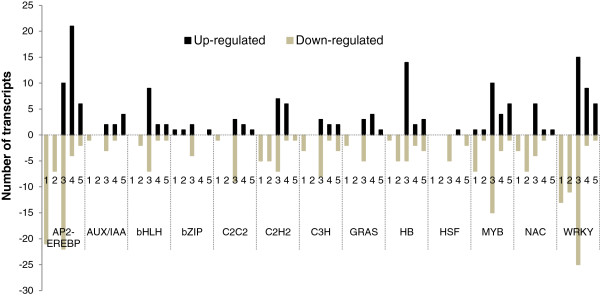
**Differentially expressed transcripts (DETs) encoding the transcription factors of various families during fibre development stages in the *fl *mutant as compared to their respective stages in WT.** 1–5 represents the 0, 5, 10, 15 and 20 dpa. Putative transcription factors at each stage are presented in the Additional file 3.

**Figure 3:**
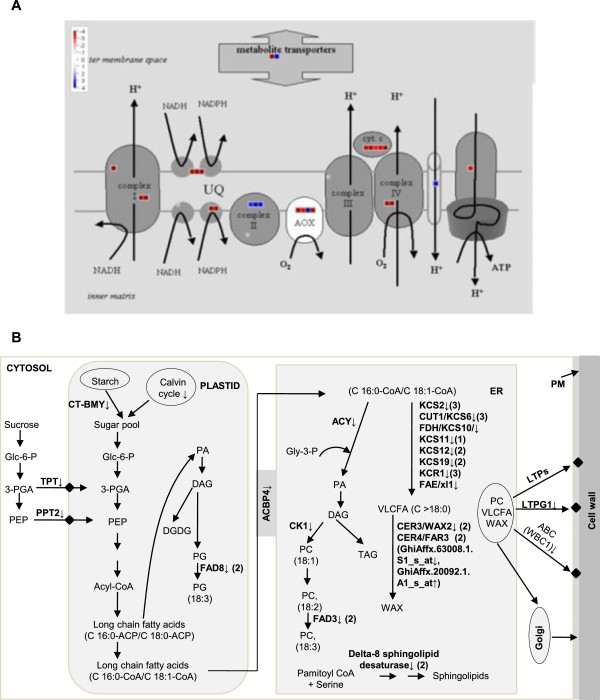
**Differentially expressed transcripts (DETs) involved in mitochondrial electron transport system (mETS) and fatty acid metabolism in the *fl *mutant as compared to WT at 10 dpa.** (**A**) MapMan-based visualization of the DETs involved in mETS in the *fl* mutant. Small blue and red colour squares represents up- and down-regulated transcripts, respectively. (**B**) Over view of DETs involved in fatty acid metabolism in the *fl* mutant. ABC: ATPase transporter, ACBP: acyl-CoA binding protein, ACY: acyltransferase-like protein, CER: eceriferum/fatty acid hydroxylase, CK: choline kinase, CT-BMY: chloroplast beta-amylase, DAG: diacylglycerol, DGDG: digalactosyldiacylglycerol, FAD: fatty acid desaturase, FAE: Long chain fatty acid elongation enzyme, FDH: Fiddlehead-like protein, Glc-6-P: glucose 6-phosphate, Gly-3-P: Glycerol-3-phosphate, KCS: beta-ketoacyl-CoA synthase, KCR: beta-ketoacyl reductase, LTP: lipid transfer protein, LTPG1: glycosylphosphatidylinositol-anchored lipid protein transfer1, PA: phosphatidic acid, PC: phosphatidylcholine, PEP: phosphoenol pyruvate, PG: phosphatidylglycerol, 3-PGA: phosphoglyceric acid, PM: plasma membrane, PPT2: phosphoenolpyruvate (PEP)/phosphate translocator 2, TAG: triacylglycerol, TPT: glucose-6-phosphate/phosphate translocator-related and VLCFA: very long chain fatty acids. DETs related to mETS and fatty acid metabolism are presented in the Additional file 6.

**Figure 4:**
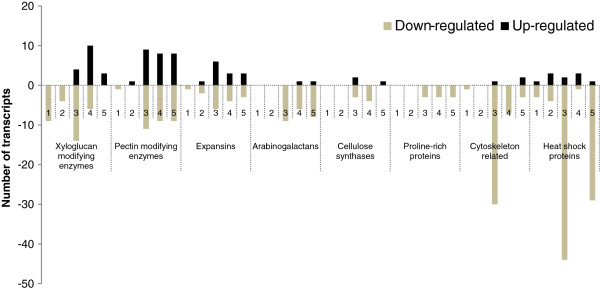
**Differentially expressed transcripts (DETs) involved in primary and secondary cell wall biosynthesis and DETs encoding the heat shock proteins (HSPs) during fibre development stages in the *fl *mutant as compared to their respective stages in WT.** Cytoskeleton related genes include ACT, TUB, ADF and actin binding proteins; Pectin modifying enzymes include PGs, PLs, PMEs and PMEIs; Xyloglucan modifying enzymes include XTHs, XETs, β-xylosidases and α-xylosidases; Expansins include EXPA, EXLA and EXLB. 1–5 represents the 0, 5, 10, 15 and 20 dpa. DETs related to cell wall biosynthesis and HSPs are presented in the Additional file 6.

The DETs were annotated based on TAIR proteome database ( http://www.arabidopsis.org). Out of 3,898 DETs, 2,968 (76.14%) were matched with *Arabidopsis* gene models with *E* value ≤ e^-10^ (Additional file 1). Further, the DETs were classified into different functional categories according to their putative functions based on MIPS functional catalogue ( http://mips.gsf.de/projects/funcat). In categories such as “protein with binding function or cofactor requirement”, “response to biotic and abiotic stresses”, “transcription”, “cellular transport, transport facilities and transport routes” and “biogenesis of cellular components” the transcripts were mostly down-regulated at fibre initiation and early elongation stages (Additional file 2). Further, DETs related to various transcription factor (TF) families and phytohormone biosynthesis and signal transduction pathways were identified using *Arabidopsis* transcription factor ( http://plntfdb.bio.uni-potsdam.de) and hormone ( http://ahd.cbi.pku.edu.cn) databases, respectively (Figure 2; Additional file 3 and Additional file 4). In addition, DETs involved in carbohydrate and energy metabolisms, fatty acid metabolism, cell wall loosening and extension and SCW synthesis were identified and discussed below (Figures 3, 4; Additional file 5 and Additional file 6).

### Validation of microarray data

**Figure 5:**
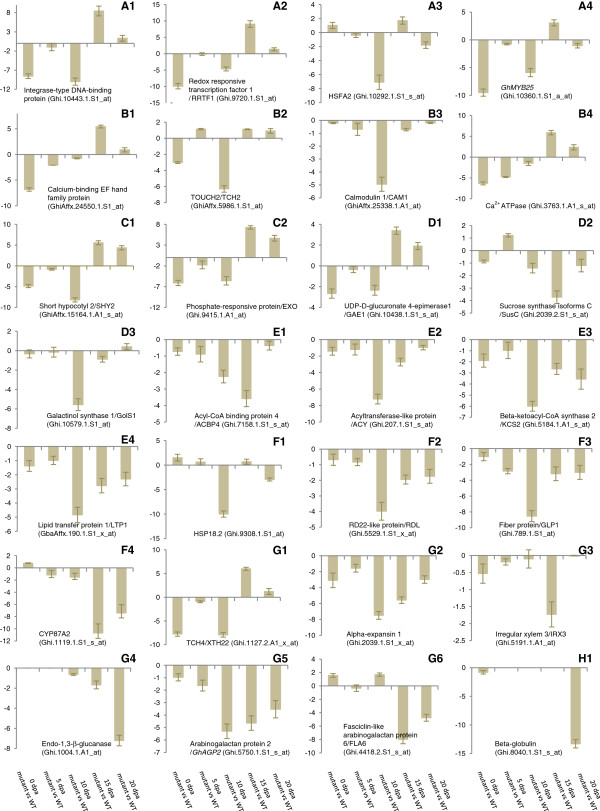
**Validation of microarray data using qRT-PCR during fibre development stages (0, 5, 10, 15 and 20 dpa) in the *fl *mutant.***Y*-axis represents the log2 fold change values at various stages in the *fl* mutant as compared to their respective stages in WT.

To validate the microarray data, quantitative real-time polymerase chain reaction (qRT-PCR) analysis was performed on 28 DETs, belonging to various functional categories, during fibre development stages (0, 5, 10, 15 and 20 dpa). The qRT-PCR analysis and microarray data showed similar gene expression pattern for all the genes studied (Figure 5, Additional file 7).

### Differentially expressed transcription factors

DETs encoding transcription factors (TFs) belonging to various families were identified in the *fl* mutant in all stages of ovule development as compared to WT (Figure 2; Additional file 3). TFs belonging to the AP2-EREBP, C2H2, NAC and WRKY were highly down-regulated at fibre initiation (0 dpa) and early elongation stages (5 dpa) in the *fl* mutant. Similarly, TFs belonging to the heat shock transcription factor (HSF) family were down-regulated at 10 and 20 dpa in the *fl* mutant (Figure 2). Further, several transcripts encoding MYB family TFs were differentially expressed at 0 and 10 dpa. In addition, transcripts encoding integrase-type DNA-binding superfamily proteins involved in defence mechanism were highly down-regulated at 0 and 10 dpa suggests their involvement in fibre development (Figure 5A1). Transcripts encoding AP2-EREBP family TFs such as ethylene responsive element binding factors (ERFs), redox responsive transcription factor 1 (RRTF1, Figure 5A2) and DREB1-like (DREBP1L) were highly down-regulated at fibre initiation (0 dpa) and elongation (5 dpa) stages in the *fl* mutant. In addition, transcripts encoding salt tolerance zinc finger (STZ) TFs belonging to C2H2-type zinc finger family and heat shock transcription factor 2 (HSFA2) were down-regulated at fibre initiation and elongation stages, respectively (Figure 5A3).

Transcripts encoding R2R3-MYB transcription factors such as *GhMYB25*/*AtMYB16* (Figure 5A4), *AtMYB106* and *AtMYB73* were down-regulated at fibre initiation and elongation stages. It has been shown that *GhMYB25*[14] and *GhMYB25like*[16] are highly expressed during fibre initiation stage and have a role in regulation of fibre cell elongation and trichome development. *AtMYB60* has been shown to be involved in root growth of *Arabidopsis* under drought stress [31]. Further, transcripts encoding NAC family TFs were down-regulated at 0 dpa (NAC047) and early elongation (NAC047, NAC072/RD26 and NAC074) stages. Another stress responsive gene, multiprotein bridging factor 1C (MBF1c) was down-regulated at 10 and 20 dpa. It has been reported that MBF1c is involved in biotic and abiotic stress tolerance by activating ethylene-responsive signal transduction pathway [32]. In addition to stress responsive TFs, transcripts encoding the homeodomain proteins such as (HOX3)/glabrous 11 and knotted1-like homeobox gene 3 (KNAT3) involved in *Arabidopsis* trichome and root development, respectively were down-regulated at 10 dpa.

The developing fibre cell during initiation and elongation stages accumulate a large amount of solutes in order to maintain the required turgor pressure. Expression of stress responsive factors during initiation and elongation stages suggests stress-like condition being generated due to turgor pressure. The stage specific transcriptome analysis showed the down-regulation of several stress responsive TFs in the *fl* mutant during the initiation and elongation stages as compared to WT. This could be due to the fact that these TFs may not have any role in the *fl* mutant as there is no further development of the fibre initials after 2 dpa. In contrast, our data showed the up-regulation of about 50% of the same TFs at much later stage (15 dpa) in the *fl* mutant (Figure 1E). The down-regulation of stress responsive TFs in the initiation stage and up-regulation of the same factors at 15 dpa in the *fl* mutant as opposed to WT suggests the loss of coordination among the regulatory mechanisms and the factors involved in the stress response in the mutant.

### Calcium and phytohormones-mediated signalling

Calcium mediated signalling plays an important role in cell division and differentiation including root hair elongation [33]. Preferential expression of genes encoding calcium binding proteins involved in Ca^2+^-mediated signalling pathways during fibre initiation and elongation stages have been reported in cotton [33,34]. In this study, several transcripts coding for calcium-binding EF-hand family proteins were highly down-regulated at 0 and 10 dpa in the *fl* mutant as compared to WT (Additional file 1; Figure 5B1, 5B2, 5B3). In addition, transcripts encoding Ca^2+^-dependent protein kinase 6 (CDPK6) and glutamate decarboxylase (GAD) were down-regulated at 0 and 10 dpa, respectively. Further, transcripts encoding Ca^2+^-ATPase that was shown to play a role in maintaining the calcium homeostasis in the cell [35] (Figure 5B4) and C2 calcium-dependent membrane targeting protein were highly down-regulated at 0 and 10 dpa in the *fl* mutant. These results are in agreement with the earlier reports that suggested a role for Ca^2+^-mediated signal transduction and Ca^2+^-homeostasis in the fibre cell initiation and elongation [33,34].

Phytohormones play an important regulatory role in various plant growth and developmental processes through intracellular signalling events leading to well-defined changes in the gene expression. In the present study, DETs involved in phytohormone biosynthesis and signal transduction pathways were identified at different stages in the *fl* mutant as compared to their respective stages in WT (Additional file 4). Ethylene [9], auxin [11] and brassinosteroids (BR) [12] were shown to play a role in fibre cell initiation and elongation. In this study genes involved in the phytohormone signal transduction pathways and the biosynthesis of auxin, BR, ethylene, gibberellic acid (GA), were found to be down-regulated at fibre initiation and elongation stages in the *fl* mutant (Additional file 4).

While the transcripts encoding cytochrome P450 83B1 (CYP83B1) were down-regulated at 0 and 5 dpa, the transcript encoding CYP79B2 was highly down-regulated at 10 dpa. CYP79B2 catalyzes the conversion of tryptophan to Indole-3-acetaldoxime and CYP83B1/RNT1/SUR2 catalyzes the first step in the biosynthesis of indole glucosinolates from indole-3-acetaldoxime and regulates the level of IAA. In addition, a transcript encoding the P-glycoprotein 19 (PGP19) involved in auxin transport was down-regulated at 10 dpa in the *fl* mutant [36]. Furthermore, transcripts encoding the auxin responsive gene hypocotyls 2 (SHY2/AUX2-11/IAA4) (Figure 5C1) were highly down-regulated at 0 and 10 dpa. Also, auxin-induced in root cultures 1 (AIR1) were highly down-regulated at 15 and 20 dpa. Transcripts encoding ACC oxidases (ACOs), involved in ethylene biosynthesis were down-regulated at 0 dpa (ACO2 and ACO4) and 5 dpa (ACO1, ACO2, ACO3 and ACO4/EFE) in the *fl* mutant. Along with ACC oxidases, a transcript encoding ACC synthase 6 (ACS6) which is a rate limiting enzyme in ethylene biosynthesis was also down-regulated at 0 dpa. These results suggest the down-regulation of auxin and ethylene biosynthesis at fibre initiation and early elongation stages in the *fl* mutant.

Transcripts encoding the enzymes involved in BR biosynthesis such as 3-oxo-5-α-steroid 4-dehydrogenase family protein (DET2) [13] and sterol methyltransferase 2 (SMT2) were down-regulated at 10 dpa in the *fl* mutant. In addition, a number of genes (eg. BRI1, BRH1 and P450-like protein/CYP734A1/BAS1) involved in BR signalling pathway [37] was also differentially expressed at various stages in the *fl* mutant as compared to WT (Additional file 4). Down-regulation of several genes involved in ethylene, auxin and BR biosynthesis and signal transduction pathways during fibre initiation and elongation stages suggested their regulatory role in fibre cell initiation and elongation. In the present study we have identified a number of genes involved in the phytohormone biosynthesis and signal transduction pathways that were down regulated at fibre initiation stage (0 dpa) in *fl* mutant as compared to WT. Interestingly, ~50% of these genes were found to be up-regulated at late elongation stage (15 dpa) in the *fl* mutant (Figure 1F), suggesting the delay in the phytohormone stimulation and signalling in the mutant.

### Carbohydrate and energy metabolism

Sugars are the basic source of energy and carbon skeletons for all biomolecules and they are required for the regulation of cell homeostasis and synthesis of cell wall precursors. UDP-D-glucose (UDP-Glc) is a central metabolite in carbohydrate metabolism and is the common precursor for synthesis of cell wall polysaccharides such as pectin, hemicellulose and cellulose. In the present study, transcripts encoding the enzymes, involved in synthesis of cell wall precursors, such as UDP-glucose pyrophosphorylases (UGP1 and UGP2), UDP-glucose 6-dehydrogenase (UGD), UDP-D-glucuronate 4-epimerase 1 (GAE1), UDP-XYL synthase 5 (UXS5) were down-regulated during fibre elongation stage in the *fl* mutant. UGP and UGD catalyse the formation of UDP-D-glucose and UDP-D-glucuronic acid, respectively whereas GAE1 (Figure 5D1) and UXS5 catalyse the formation of UDP-D-galacturonic acid (precursor for pectin) and UDP-D-xylose (precursor for hemicellulose) from UDP-D-glucuronic acid, respectively (Additional file 5 and Additional file 6). The UDP-glucose formed from the sucrose by sucrose synthase (Sus) is used directly as a substrate by the cellulose synthase complex. The Sus isoform C (SusC) involved in cellulose synthesis was highly down-regulated at 15 dpa in the *fl* mutant (Figure 5D2, Additional file 5 and Additional file 6) [38]. Carbohydrates such as raffinose family oligosaccharides (RFOs) are the main storage forms of carbohydrates in the seeds, which confer desiccation tolerance. In the present study, transcripts encoding galactinol synthase (GolS) that catalyse the first committed step in the biosynthesis of RFOs (GolS1 and GolS2) were highly down-regulated at 10 dpa in the *fl* mutant (Figure 5D3; Additional file 5 and Additional file 6). Further, raffinose synthase (RS/SIP1), stachyose synthase (STS) were also down-regulated. Trehalose is another storage carbohydrate that was shown to be involved in desiccation tolerance [39]. Transcripts encoding the enzymes involved in trehalose biosynthesis such as trehalose-6-phosphate synthase (TPS) and trehalose-6-phosphate phosphatase (TPP) were down-regulated at fibre elongation stage. Previously we have shown that, both GolS and TPS were highly up-regulated under drought stress at fibre elongation stage suggesting the role of these genes in fibre development [40].

In the present study, transcripts encoding the enzymes involved in Calvin cycle such as ribulose bisphosphate carboxylase, glyceraldehyde 3-phosphate dehydrogenase, transketolase and fructose-bisphosphate aldolase (FBPA) were down-regulated at 10 and 15 dpa thus indicating the reduction of sugar pool in the *fl* mutant ovules (Additional file 6). Mitochondrial electron transport system (mETS) is the main pathway for the generation of ATP by oxidative phosphorylation. Recently it has been shown that the ATP synthase δ1 subunit (*GhATPδ1*) is required for mitochondrial ATP synthesis and to maintain higher ATP⁄ADP ratio which facilitates faster fibre cell elongation [21]. Several transcripts encoding the enzymes involved in mETS were down-regulated in the *fl* mutant which in turn decreases the NAD^+^/NAD(P)H ratio and efficiency of mitochondrial ATP production (Figure 3A; Additional file 6). The ATP/ADP and NAD^+^/NAD(P)H ratios are very critical for maintaining redox-homeostasis and functioning of several enzymes involved in synthesis of cell wall precursors and fatty acids [41]. These data suggest the importance of interconversion of sugars and the role of mETS during fibre elongation process.

### Fatty acid metabolism

Fatty acid biosynthesis is another important biochemical pathway involved in fibre cell development [5,42,43]. In the present study, transcripts encoding the enzymes involved in the biosynthesis of very long chain fatty acids (VLCFAs), cuticular wax and phospholipids were down-regulated at fibre elongation stage in the *fl* mutant as compared to WT. Calvin cycle and sugars exported from the cytosol are main source of sugar pool for synthesis of fatty acids in plastids of non-photosynthetic tissue. Fatty acids synthesized within the plastids are exported as acyl-CoA esters (C16:0 CoA and C18:1 CoA) to the endoplasmic reticulum (ER) for synthesis of VLCFA that are precursors for synthesis of phospholipids, sphingolipids, wax and cuticular matrix (Figure 3B; Additional file 6). Transcripts encoding the sugar transporters such as triose phosphate/phosphate translocator (TPT) and phosphoenolpyruvate (PEP)/phosphate translocator (PPT) and acyl-CoA binding protein 4 (ACBP4, Figure 5E1) were down-regulated at 10 dpa in the *fl* mutant. Glucose-6-phosphate/phosphate translocator (GTP), TPT and PPT are required for import of cytosolic Glc-6-P, 3-PGA and PEP respectively, from cytosol into the plastid for synthesis of acyl-CoA [44]. Cytosolic ACBP4 and ACBP5 were shown to bind long-chain acyl-CoAs and involved in the trafficking of oleoyl-CoA from the plastids to ER for synthesis of VLCFAs [45].

Phospholipids are major structural components of plasma membrane (PM) and involved in lipid signalling pathway. PM plays a crucial role during fibre cell elongation. Phosphatidic acid (PA) is the key component in phospholipid biosynthesis. The transcripts encoding acyltransferase-like protein (ACY) involved in synthesis of PA in ER (Figure 5E2) and AGC (cAMP-dependent, cGMP-dependent and protein kinase C) kinase family proteins (AGC2-1 and AGC2-2) were highly down-regulated at 10 dpa in the *fl* mutant. Further, transcripts encoding fatty acid desaturases (FADs) such as FAD8 (plasticidal) and FAD3 (ER) and delta-8 sphingolipid desaturase were down-regulated. In lipid-signalling pathway PA specifically binds to *AtPDK1* (3-phosphoinositide-dependent protein kinase 1) and stimulates AGC2-1 and it has been shown that agc2-1 knockout mutants resulted in reduced root hair length suggesting its role in cell elongation [46]. Down-regulation of genes involved in phospholipid biosynthesis and lipid signalling suggested their role in fibre development.

Transcripts encoding beta-keto acyl-CoA synthases (KCS2, KCS6/CER6/CUT1, KCS10/FDH, KCS11, KCS12 and KCS19) and beta-keto acyl reductase (KCR1/YBR159), catalyzing the first two committed steps in VLCFA synthesis, were highly down-regulated at fibre elongation stage in the *fl* mutant (Figures 3B, 5E3). In addition, a transcript encoding a long chain fatty acid elongation enzyme (ELo2/SUR4) was highly down-regulated at 5, 10 and 20 dpa. A transcript encoding epoxide hydrolase involved in cutin layer biosynthesis was highly down-regulated at 10 dpa [47].

Lipid transfer proteins (LTPs) are involved in transport of lipids from ER to PM and the subsequent transport of lipids from PM to the cell exterior appears to be carried out by PM localized transporters such as ATP-binding cassette (ABC) transporters [48,49]. In the present study, several transcripts encoding LTPs (Figure 5E4), ABC transporters such as white/brown complex proteins (WBC1 and WBC12/CER5) were down-regulated at fibre elongation stage in the *fl* mutant. Further, a transcript encoding glycosylphosphatidylinositol-anchored lipid protein transfer 1 (LTPG1) was down-regulated. High level expression of WBC1 during fibre elongation and plasma membrane localization was reported in upland cotton [50]. In *A*. *thaliana* WBC12/CER5 and WBC11 have been shown to be involved in cuticular lipid transport [51] and the localization of LTPG in PM and its role in lipid deposition has been demonstrated [52]. Down-regulation of genes involved in fatty acid metabolism in the *fl* mutant particularly at elongation stage suggests the importance of these genes in fibre cell development.

### Transcripts involved in regulation of osmotic potential, ion-homeostasis and protein stabilization

Cotton fibre elongates through diffuse growth mechanism and turgor-driven pressure is a significant driving force for cell enlargement [17,18,53,54]. The turgor-driven pressure largely depends on accumulation of osmolytes and ion-homeostasis [17,18]. Further, genes involved in ROS homeostasis and protein stabilization play a crucial role during fibre development [6,8]. In the present study several transcripts involved in these processes were differentially expressed at various fibre development stages in the *fl* mutant as compared to WT. Transcripts encoding the ion and sugar transporters (NRT1.5, VIT and STP1) and membrane intrinsic proteins (PIP2;2 and γ-TIP1;3) were highly down-regulated during fibre elongation stage in the *fl* mutant (Table 1, Figure 5F15F4). A transcript encoding SKD1 (suppressor of K^+^ transport growth defect 1) which is an AAA-type ATPase family protein was highly down-regulated at 10 dpa in the *fl* mutant. The expression of *mcSKD1* (homologous to *AtSKD1*) in elongating root tips and its role in endoplasmic reticulum-Golgi mediated protein sorting machinery and K^+^ uptake has been demonstrated [55]. Further, transcripts encoding heat shock proteins (sHSPs, HSP70 and HSP90) were highly down-regulated at 10 and 20 dpa in the *fl* mutant.

**Table 1:** **Some of the differentially expressed stress responsive transcripts at various fibre development stages in the*fl*mutant as compared to their respective stages in WT**

**Probe set ID**	**0 dpa**	**5 dpa**	**10 dpa**	**15 dpa**	**20 dpa**	**Annotation**
Ghi.10292.1.S1_s_at			−108.2		−6.7	Heat shock transcription factor A2 (HSFA2)
Ghi.9308.1.S1_at	4.2		−142.5		−6.5	Heat shock protein HSP18.2
GhiAffx.3815.1.A1_s_at			−62.9		−10.9	HSP90
Ghi.8378.2.S1_s_at			−36.1		−5.8	HSP70
GhiAffx.10920.2.S1_at		−11.9				Plasma membrane intrinsic protein 2;2 (PIP2;2)
Ghi.4613.1.S1_at			−36.5	−6.7		Gamma-tonoplast intrinsic protein 1;3 (γ-TIP1;3)
Ghi.3135.1.S1_at		−62.5				Nitrate transporter 1.5 (NRT1.5)
Ghi.6855.1.A1_s_at		−6.5				Proline transporter 2 (PROT2)
Ghi.9885.1.A1_at		−4.6				Sugar transporter 1 (STP1)
GhiAffx.17156.1.S1_at			−22.7			Vacuolar iron transporter (VIT)
Ghi.10646.1.S1_s_at		−5.2	178.7		102.2	Osmotin 34
GarAffx.24961.2.S1_s_at	−3.3		−22.3	−8		Responsive to desiccation 22 (RD22)
Gra.2810.2.A1_s_at	−3.2		−21.8	−8.2		RD22
Ghi.5529.1.S1_x_at	−3		−35	−8.2	−4.4	RD22-like protein (RDL)
Ghi.779.1.S1_at		−20.5				Glutathione S-transferase 30 (GST30)
Ghi.7950.1.S1_at		−14				Peroxidase 2 (PA2)
Ghi.10450.1.S1_s_at	−4.8		−13.3	7		Thioredoxin family protein (GRX480)
Gra.2231.1.S1_s_at	−13.2	−26.2	27.4			Asparagine synthase 1 (ASN1)
Ghi.789.1.S1_at			−147.1	−21.3	−10.5	Germin-like protein (GLP1)/Fibre protein
Ghi.6103.1.S1_at			−28.8	−9.4	−3.2	Chalcone synthase (CHS)
Gra.432.1.S1_s_at	−19.8	−5.1		−4.6	17.7	CHS
Ghi.3763.1.A1_s_at	−52.9	−16.5		28.4	3.7	Ca^2+^-ATPase
GhiAffx.3680.3.A1_at			−34.4			Suppressor of K^+^ transport growth defect-like protein (SKD1)

### Primary and secondary cell wall biosynthesis

Several transcripts involved in the primary and secondary cell wall biosynthesis were differentially expressed at various stages of fibre development in the *fl* mutant as compared to their respective stages in WT (Figure 4; Additional file 6). A large number of genes involved in primary cell wall biosynthesis and elongation such as those coding for xyloglucanases, pectinases, expansins and arabinogalactans were down-regulated at 15 dpa. The role of xyloglucan modifying enzymes such as xyloglucan endotransglycosylases (XETs) and xyloglucan endotransglucosylase/hydrolases (XTHs) has been well established in fibre cell development [3,22]. In the present study, transcripts encoding TOUCH 4 (TCH4)/XTH22 (Figure 5G1) and XTH23/XTR6 were highly down-regulated at initiation and elongation stages in the *fl* mutant. Further, transcripts encoding endo-xyloglucan transferase/*AtXTH7* involved in fibre elongation were highly down-regulated at 10 and 15 dpa. These data suggested their role in primary cell wall synthesis and fibre development. Pectins are major components of the primary cell wall and in cotton pectins constitute about 25% of the cell wall of cotton fibre. Thus, pectin modifying enzymes play a major role in the fibre cell wall development [56]. Several transcripts encoding pectin modifying enzymes such as poly galacturonases (PGs), pectate lyases (PLs), pectin methyl esterases (PMEs) and pectin methyl esterase inhibitors (PMEIs) were differentially expressed during fibre elongation stage in the *fl* mutant as compared to WT (Figure 4; Additional file 6). Further, large number of transcripts encoding glycosyl hydrolases and transferases were down-regulated at 10 and 15 dpa. Particularly, transcripts encoding β-galactosidase 13 (BGAL13) and UDP-glucosyl transferase 74B1 (UGT74B1) were highly down-regulated at 10 dpa in the *fl* mutant. Among the glycosyl hydrolases, galactosidases (GALs) catalyzes the removal of non-reducing β-D-galactosyl residues from β-D-galactosides. It is therefore thought that GALs may be one of the main enzymes responsible for the metabolism of galactose rich polymers such as galactan and arabinogalactan and galactose containing side chains of cell wall polysaccharides [3,5].

Expansins are highly expressed in cotton fibre tissue and play an important role in cell wall loosening during fibre elongation stage [3,24]. A transcript (Ghi.2039.1.S1_x_at/AY189969.1) encoding α-expansin 1 (EXPA1) was highly down-regulated at 10, 15 and 20 dpa in the *fl* mutant (Figure 5G2; Additional file 6). Further, transcripts encoding expansin-like B1 (EXLB1) were highly down-regulated at 0 and 5 dpa and expansin-like α (EXLA) were down-regulated at 10 dpa. These data suggest that both expansins and expansin-like genes play crucial role in fibre cell development. The transcripts encoding cellulose synthases were down-regulated at 15 dpa at which PCW synthesis ceases and cellulose synthesis begins (Figure 5G3; Additional file 6). Further, a transcript encoding endo-1,3-β-glucanase was highly down-regulated at 20 dpa in the *fl* mutant (Figure 5F4). It was reported that the expression of endo-1,3-β-glucanase, which is very low at the fibre elongation stage (5–12 dpa) and increases gradually during secondary cell wall formation in *G*. *hirsutum* (15–20 dpa), is involved in the degradation of callose and opening of the plasmodesmata during cellulose deposition [5,19]. Interestingly, a transcript encoding trichome birefringence (TBR) like 38 (TBL38) gene was highly down-regulated at 5 dpa and 15 dpa. Recently, it has been shown that TBL genes are involved in secondary cell wall synthesis by influencing the esterification state of pectic polymers and increasing the crystalline cellulose in *Arabidopsis*[57]. Along with the cell wall modifying enzymes, several transcripts encoding structural proteins such as AGPs (*GhAGP2*), FLAs (*GhFLA6*/*AtFLA11* and *GhFLA3*/*AtFLA7*) and proline-rich proteins (PRPs; PRP3 and PRP5) were down-regulated at fibre elongation and SCW synthesis stages in the *fl* mutant (Figures 4, 5G5, 5G6; Additional file 6).

Actin cytoskeleton plays an important role in fibre elongation and onset of secondary cell wall deposition [6,26,27]. Tubulins (TUBs) are major components of cytoskeleton microtubules and play a vital role in cell expansion by controlling the orientation of cellulose microfibrils [25]. In the present study, transcripts encoding actins (ACTs), profilin, ATP binding microtubule motor family proteins, actin depolymerizing factor (ADF) and Xu-142 beta-tubulin1 were down-regulated at 10 dpa in the *fl* mutant (Figure 4, Additional file 6). The down-regulation of several genes involved in cell wall loosening and elongation, structural reinforcement and cytoskeleton dynamics suggests their role in fibre development.

## Conclusions

SEM study indicated a delay in the initiation process of fibre cells on the epidermal layer of ovules in the *fl* mutant and elongation of these initials was completely stopped at about 2 dpa. Transcriptome analysis revealed the differentially expressed transcripts at various fibre developmental stages (0, 5, 10, 15 and 20 dpa) in the *fl* mutant as compared to their respective stages in the wild-type. The down-regulation of stress responsive TFs (representing 19.6% of total down-regulated transcripts) and transcripts involved in the Ca^2+^-mediated signal transduction and phytohormone (ethylene, auxin and BR) biosynthesis and signalling pathways at fibre initiation stage suggested their regulatory role in fibre cell initiation and differentiation. Further, down-regulation of transcripts involved in the Calvin cycle, synthesis of RFOs (GolS1, GolS2), trehalose (TPS and TPP) and cell wall precursors and mitochondrial electron transport system at fibre elongation stage in the *fl* mutant suggested the reduction of overall sugar pool and significance of mitochondrial energy supply required for the synthesis of cell wall polysaccharides and to maintain redox-homeostasis. Similarly, down-regulation of transcripts involved in the synthesis of VLCFAs and phospholipids and lipid transport suggests the reduced fatty acid metabolism in the *fl* mutant. This study also supports the earlier findings on the role of pectin and xyloglucan modifying enzymes, expansins and arabinogalactans and cytoskeleton dynamics in the cell wall loosening and elongation process. Also the delayed expression of genes involved in phytohormone signalling and stress responsive TFs identified in the present study suggests the lack of coordinated expression of regulatory mechanisms involved in fibre cell initiation and differentiation in *fl* mutant. On the whole, the comprehensive transcriptome analysis of *Gossypium hirsutum* L. cv. MCU5 and its near isogenic fuzzless-lintless mutant revealed the stage specific involvement of several genes and pathways during fibre development.

## Methods

### Plant material

The *Gossypium hirsutum* L. cv. MCU5 (wild-type) and its near isogenic fuzzless-lintless mutant were grown in the field following normal agronomic practices. Flowers were tagged on the day of anthesis and considered as 0 dpa (days post anthesis). Cotton bolls were collected at fibre initiation (−2, -1, 0, 1 and 2 dpa), elongation (5, 10 and 15 dpa) and secondary cell wall synthesis (20 dpa) stages. The −2 and −1 dpa represent the days before anthesis. Harvested cotton bolls were immediately frozen in liquid nitrogen and stored at −70°C until used for total RNA extraction.

### Scanning electron microscopy analysis

Scanning electron microscopy (SEM) was performed at fibre initiation stage (−2 to 2 dpa) to observe the development of fibre initials on epidermal layer of ovules as described by Mir and Channa [58]. SEM was carried out with LEO 435 VP scanning electron microscope (LEO Electron Microscopy Ltd., Cambridge, England) at All India Institute of Medical Sciences (AIIMS), New Delhi, India.

### RNA isolation

Total RNA was isolated from fibre-bearing ovules of wild-type (WT) and fuzzless-lintless (*fl*) mutant collected at various stages of fibre development (0, 5, 10, 15 and 20 dpa). RNA was isolated using Spectrum^TM^ Plant Total RNA kit (Sigma, USA) according to the manufacturer’s protocol. During RNA purification on-column DNase treatment was given for removing trace amount of DNA. The quality and quantity of total RNA was assessed by Agilent Bioanalyzer 2100 (Agilent Technologies, USA).

### Microarray experiments and data analysis

Affymetrix cotton GeneChip genome array (Affymetrix, USA) having 23,977 probe sets representing 21,854 cotton transcripts was used for transcriptome analysis. Three biological replicates were used to test the reproducibility and quality of the chip hybridization. The One-Cycle Target Labeling and Control Reagent kit was used to prepare biotinylated complementary RNA (cRNA) for microarray hybridization. Array hybridization, staining and washing procedures were carried out as described in the Affymetrix protocols. The arrays were scanned with a GeneChip scanner 3000.

DAT, CEL, CHP, XML and JPEG image files were generated for each array using GeneChip Operating Software (GCOS) platform. CEL files having estimated probe intensity values were analyzed with GeneSpring GX-11.5 software (Agilent Technologies, USA) to get differentially expressed transcripts. The Robust Multiarray Average (RMA) algorithm was used for the background correction, quantile normalization and median polished probe set summarization to generate single expression value for each probe set. Normalized expression values were log2-transformed and differential expression analysis was performed using unpaired *t*-test. The *p* values were corrected by applying the false discovery rate (FDR) correction [59]. Differentially expressed transcripts with FDR corrected *p* value ≤ 0.01 and fold change ≥ 3 were included for further data analysis. The DETs were annotated based on NetAffx annotation data for cotton GeneChip ( http://www.affymetrix.com). To obtain functional annotation of transcripts, the consensus sequences of probe sets present in the cotton GeneChip genome array were mapped to the Arabidopsis TAIR protein database version 10 ( http://www.arabidopsis.org) by BLASTX with *E* value cut off ≤ e^-10^. Further, the DETs were grouped into various functional categories by using their corresponding *Arabidopsis* protein IDs in MIPS functional catalogue ( http://mips.gsf.de/projects/funcat). To identify the putative transcription factors and transcripts related to phytohormone biosynthesis and signal transduction pathways, the consensus sequences of all probe sets presented in cotton GeneChip genome array were searched against the Arabidopsis transcription factor database ( http://plntfdb.bio.uni-potsdam.de, version 3.0) and Arabidopsis hormone database ( http://ahd.cbi.pku.edu.cn, version 2.0) respectively, by BLASTX with *E* value cut-off ≤ e^-10^. Further, MapMan software version 3.5.0 was used to visualize the expression of differentially regulated cotton transcripts onto metabolic pathways ( http://gabi.rzpd.de/projects/MapMan/). The microarray data is deposited in the Gene Expression Omnibus database (GEO, http://www.ncbi.nlm.nih.gov/geo) at the NCBI under the Series Accession numbers GSE38490. The hierarchical clustering was performed based on centroid linkage with Euclidean distance using log fold change data with Cluster 3.0 to display the expression pattern and tree diagram of differentially expressed transcripts [60].

### The quantitative real-time PCR (qRT-PCR) analysis

First strand cDNA was synthesized using 1 μg of total RNA using AffinityScript QPCR cDNA Synthesis Kit (Stratagene, Agilent Technologies, USA) according to the manufacturer’s instructions. Gene specific qRT-PCR primers were designed using PrimerQuest software ( http://eu.idtdna.com) and the list of primers are presented in Additional file 7. The qRT-PCR was performed in triplicates using the Brilliant-III Ultra Fast SYBR Green QPCR master mix in Stratagene MX 3005P (Agilent Technologies, USA) detection system. The *GhPP2A1* gene (Accession No: DT545658) from *G*. *hirsutum* was used as reference gene to normalize the expression values [61]. The log2 fold change value was calculated based on 2^–(∆∆Ct)^ method.

## Competing interests

The authors declare that they have no competing interests.

## Authors’ contribution

KVP carried out microarray experiments, analyzed the transcriptome data and drafted the manuscript. KK carried out designing of primers, qRT-PCR analysis and revise the manuscript. GD, MK and MLVP and NJ conducted microarray experiments. SK and DPP involved in the SEM studies. AHP was involved in the SEM and manuscript preparation. TCM, HV and ISK planned and executed experiments to develop stage specific materials and collected stage specific samples for the transcriptome analysis and contributed in the manuscript preparation. VSR, PAK, SL and MKR involved in the designing of the experiments, data analysis, manuscript preparation and discussion. All authors read and approved the manuscript.

## Supplementary Material

Additional file 1 **List of differentially expressed transcripts (DETs) at 0, 5, 10, 15 and 20 dpa in the *fl* mutant as compared to their respective stages in WT.** Excel file containing the DETs with p value ≤ 0.01 and fold change ≥ 3. Click here for file

Additional file 2 **Functional classification of DETs at 0, 5, 10, 15 and 20 dpa in the *fl *mutant as compared to their respective stages in WT.** PPT file containing the DETs grouped into various functional categories based on MIPS data base. Click here for file

Additional file 3 **List of DETs encoding putative transcription factors (TFs) belonging to various families at 0, 5, 10 and 20 dpa in the *fl *mutant as compared to their respective stages in WT.** Excel file containing the differentially expressed putative transcription factors based on Arabidopsis transcription factor database (*E*-value cutoff ≤ e^-10^). Click here for file

Additional file 4 **List of DETs involved in phytohormone biosynthesis and signalling pathways at 0, 5, 10, 15 and 20 dpa in the *fl *mutant as compared to their respective stages in WT.** Excel file containing the DETs involved in phytohormone biosynthesis and signalling pathways were identified based on Arabidopsis hormone database (*E* value cutoff ≤ e^-10^). (XLS 669 kb) Click here for file

Additional file 5: **Differentially expressed transcripts (DETs) involved in carbohydrate metabolism at 0, 5, 10, 15 and 20 dpa in the *fl *mutant as compared to their respective stages in WT.** PPT file containing the DETs involved in biosynthesis of RFO, trehalose and cell wall precursors. Click here for file

Additional file 6 **List of DETs related to fatty acid metabolism, carbohydrate and energy metabolism, cell wall loosening and elongation 0, 5, 10, 15 and 20 dpa in the *fl *mutant as compared to their respective stages in WT.** Excel file containing the differentially expressed transcripts related to different functional categories based on MIPS and MapMan databases. Click here for file

Additional file 7 **List of primers used for qRT-PCR analysis.** Excel file containing the primer sequences used for qRT-PCR to validate microarray data. Click here for file
