# Supplementary material for: Functional genomics of fuzzless-lintless mutant of Gossypium hirsutum L. cv. MCU5 reveal key genes and pathways involved in cotton fibre initiation and elongation
Source: BMC Genomics. 2012 Nov 14;13:624. doi: 10.1186/1471-2164-13-624 (PMC3556503; doi:10.1186/1471-2164-13-624)
Supplement: Additional file 2 — Functional classification of DETs at 0, 5, 10, 15 and 20 dpa in the fl mutant as compared to their respective stages in WT. PPT file containing the DETs grouped into various functional categories based on MIPS data base. [file 1471-2164-13-624-S2.ppt]

## Slide 1
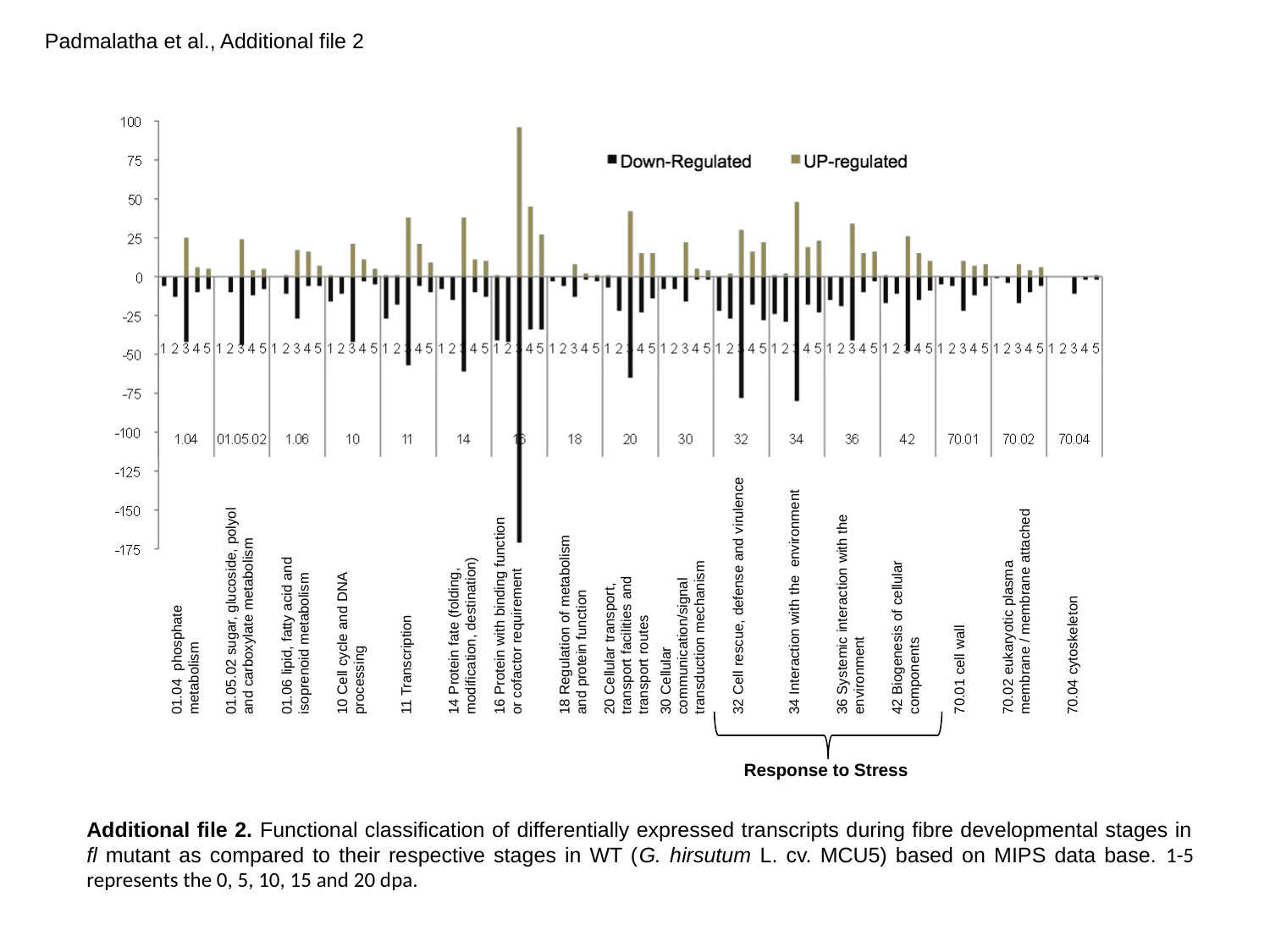

Padmalatha et al., Additional file 2
32 Cell rescue, defense and virulence
34 Interaction with the environment
14 Protein fate (folding, modification, destination)
01.05.02 sugar, glucoside, polyol and carboxylate metabolism
70.02 eukaryotic plasma membrane / membrane attached
20 Cellular transport, transport facilities and transport routes
30 Cellular communication/signal transduction mechanism
01.06 lipid, fatty acid and isoprenoid metabolism
10 Cell cycle and DNA processing
36 Systemic interaction with the environment
42 Biogenesis of cellular components
16 Protein with binding function or cofactor requirement
70.01 cell wall
70.04 cytoskeleton
18 Regulation of metabolism and protein function
11 Transcription
01.04 phosphate metabolism
Response to Stress
Additional file 2. Functional classification of differentially expressed transcripts during fibre developmental stages in fl mutant as compared to their respective stages in WT (G. hirsutum L. cv. MCU5) based on MIPS data base. 1-5 represents the 0, 5, 10, 15 and 20 dpa.
