# Supplementary material for: Functional genomics of fuzzless-lintless mutant of Gossypium hirsutum L. cv. MCU5 reveal key genes and pathways involved in cotton fibre initiation and elongation
Source: BMC Genomics. 2012 Nov 14;13:624. doi: 10.1186/1471-2164-13-624 (PMC3556503; doi:10.1186/1471-2164-13-624)
Supplement: Additional file 5: — Differentially expressed transcripts (DETs) involved in carbohydrate metabolism at 0, 5, 10, 15 and 20 dpa in the fl mutant as compared to their respective stages in WT. PPT file containing the DETs involved in biosynthesis of RFO, trehalose and cell wall precursors. [file 1471-2164-13-624-S5.ppt]

## Slide 1
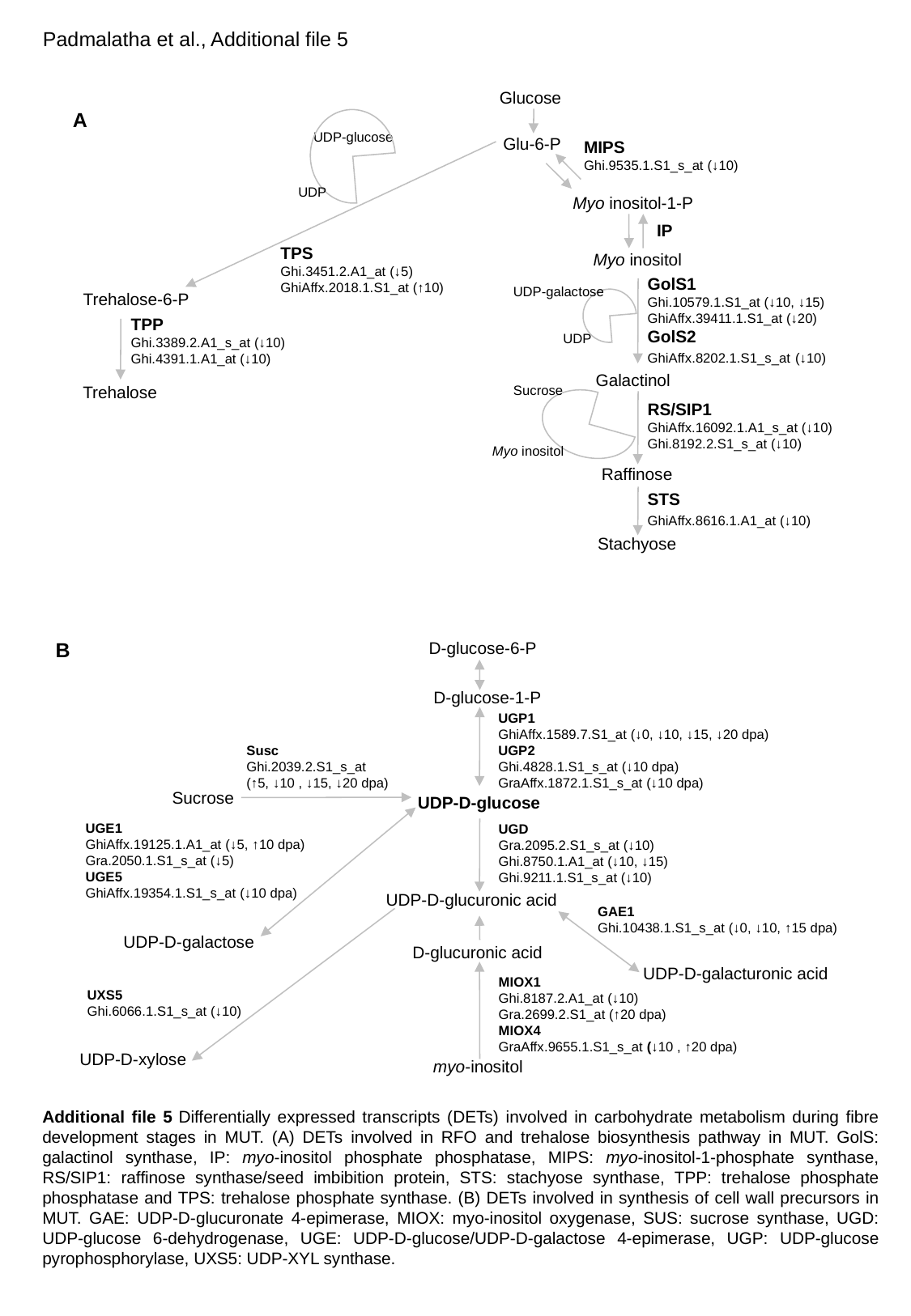

Padmalatha et al., Additional file 5
Glucose
A
UDP-glucose
Glu-6-P
MIPS
Ghi.9535.1.S1_s_at (↓10)
UDP
Myo inositol-1-P
IP
TPS
Ghi.3451.2.A1_at (↓5)
GhiAffx.2018.1.S1_at (↑10)
Myo inositol
GolS1
Ghi.10579.1.S1_at (↓10, ↓15)
GhiAffx.39411.1.S1_at (↓20)
GolS2
GhiAffx.8202.1.S1_s_at (↓10)
UDP-galactose
Trehalose-6-P
TPP
Ghi.3389.2.A1_s_at (↓10)
Ghi.4391.1.A1_at (↓10)
UDP
Galactinol
Trehalose
Sucrose
RS/SIP1
GhiAffx.16092.1.A1_s_at (↓10) Ghi.8192.2.S1_s_at (↓10)
Myo inositol
Raffinose
STS
GhiAffx.8616.1.A1_at (↓10)
Stachyose
B
D-glucose-6-P
D-glucose-1-P
UGP1
GhiAffx.1589.7.S1_at (↓0, ↓10, ↓15, ↓20 dpa)
UGP2
Ghi.4828.1.S1_s_at (↓10 dpa)
GraAffx.1872.1.S1_s_at (↓10 dpa)
Susc
Ghi.2039.2.S1_s_at
(↑5, ↓10 , ↓15, ↓20 dpa)
Sucrose
UDP-D-glucose
UGE1
GhiAffx.19125.1.A1_at (↓5, ↑10 dpa)
Gra.2050.1.S1_s_at (↓5)
UGE5
GhiAffx.19354.1.S1_s_at (↓10 dpa)
UGD
Gra.2095.2.S1_s_at (↓10)
Ghi.8750.1.A1_at (↓10, ↓15)
Ghi.9211.1.S1_s_at (↓10)
UDP-D-glucuronic acid
GAE1
Ghi.10438.1.S1_s_at (↓0, ↓10, ↑15 dpa)
UDP-D-galactose
D-glucuronic acid
UDP-D-galacturonic acid
MIOX1
Ghi.8187.2.A1_at (↓10)
Gra.2699.2.S1_at (↑20 dpa)
MIOX4
GraAffx.9655.1.S1_s_at (↓10 , ↑20 dpa)
UXS5
Ghi.6066.1.S1_s_at (↓10)
UDP-D-xylose
myo-inositol
Additional file 5 Differentially expressed transcripts (DETs) involved in carbohydrate metabolism during fibre development stages in MUT. (A) DETs involved in RFO and trehalose biosynthesis pathway in MUT. GolS: galactinol synthase, IP: myo-inositol phosphate phosphatase, MIPS: myo-inositol-1-phosphate synthase, RS/SIP1: raffinose synthase/seed imbibition protein, STS: stachyose synthase, TPP: trehalose phosphate phosphatase and TPS: trehalose phosphate synthase. (B) DETs involved in synthesis of cell wall precursors in MUT. GAE: UDP-D-glucuronate 4-epimerase, MIOX: myo-inositol oxygenase, SUS: sucrose synthase, UGD: UDP-glucose 6-dehydrogenase, UGE: UDP-D-glucose/UDP-D-galactose 4-epimerase, UGP: UDP-glucose pyrophosphorylase, UXS5: UDP-XYL synthase.
